# Low‐to‐Moderate Daytime Physical Activities Predicted Higher‐Quality Sleep Among Habitually Active Agropastoralists

**DOI:** 10.1002/ajhb.70008

**Published:** 2025-02-06

**Authors:** Ming Fei Li, Puseletso Lecheko, Tumelo Phuthing, Tsepo Lesholu, David R. Samson

**Affiliations:** ^1^ Department of Anthropology University of Toronto Toronto Ontario Canada; ^2^ Mehloding Community Tourism Trust Matatiele Eastern Cape South Africa; ^3^ Department of Anthropology University of Toronto Mississauga Mississauga Ontario Canada

**Keywords:** actigraphy, agropastoral, physical activity, rural, sleep

## Abstract

**Introduction:**

The positive effects of physical activity (PA) on sleep are widely promoted by public health organizations and supported by abundant empirical evidence. Nonetheless, there remains a dearth of studies investigating the association between daytime PA and nighttime sleep among non‐urban and nonindustrial populations that habitually engage in PA as part of their subsistence strategy.

**Methods:**

Here, we examined the bidirectional relationship between PA and sleep. We also looked at age, gender, and occupation‐level differences in moderate‐to‐vigorous‐intensity PA (MVPA), low‐intensity PA (LPA), and sedentary activity durations among Basotho and Xhoxa agropastoralists residing in rural villages in the Eastern Cape, South Africa. We analyzed activity and sleep data collected from 113 individuals using MotionWatch actigraphy wristwatches across three field seasons (7111 individual days).

**Results:**

Percentage daily total MVPA decreased with age, though older participants maintained low activity levels and did not suffer from poorer sleep compared to younger participants. Herders spent more percentage of their day in higher‐intensity activity than non‐herders. Overall, women had greater daily percentage MVPA and lower percentage sedentary activity than men. Durations of total MVPA and LPA decreased total sleep time (TST) and improved sleep quality (increased sleep efficiency (SE), decreased fragmentation, and decreased percentage wake after sleep onset). Daytime PA measures were not affected by sleep duration or quality from the previous night.

**Conclusions:**

Among this group of habitually active agropastoralists, low‐to‐moderate‐intensity PA durations consistently predicted higher sleep quality. Our findings showed that sleep quality was more strongly affected by PA than sleep duration.

## 1 Introduction

Sleep facilitates healthy physiological and cognitive functioning through processes such as energy optimization (Schmidt 2014), cellular recovery (Mignot 2008), emotional regulation (Walker 2009), and memory consolidation (Stickgold 2005). Inadequate sleep can lead to poor physical and mental health outcomes (Luyster et al. 2012). A widely accepted non‐pharmacological intervention for improving sleep and overall well‐being is engagement in physical activity (PA) (Kline et al. 2021). Populations in developed and developing countries are spending increasingly more time in sedentary behaviors (e.g., working at a desk, watching TV, sitting in a vehicle), which has raised global public health concerns (Owen et al. 2020). National and international health organizations have published guidelines to ensure sufficient PA levels are met for promoting better health (e.g., Physical Activity Guidelines for Americans, 2nd Edition, 2018), and these guidelines repeatedly emphasize the link between PA and sleep (Kline et al. 2021).

Studies using human and animal models have allowed us to have a clearer understanding of the possible underlying physiological mechanisms through which PA positively influences sleep (reviewed in Uchida et al. 2012). For instance, daytime PA can lead to increased heart rate (HR) during sleep (Uchida et al. 2012) and regular exercise has been shown to increase vagal modulation, measured as increased heart rate variability (HRV); increased HRV enhances parasympathetic control, which can improve mood and sleep (Uchida et al. 2012). Daytime PA can impact sleep via endocrine processes as well (Uchida et al. 2012). A recent review found that PA decreased cortisol levels (i.e., stress response) and improved subjective sleep quality (De Nys et al. 2022). Cortisol is a hormone secreted in response to stress, and it follows a circadian rhythmicity pattern characterized by an initial peak upon awakening followed by a gradual decline throughout the day until a low point around midnight or bedtime (reviewed in De Nys et al. 2022). Decreased cortisol levels before bedtime can reduce sleep disruptions and contribute to better sleep (De Nys et al. 2022). Additionally, we know that PA can elicit positive changes in mood and mental health (Penedo and Dahn 2005; Zapalac et al. 2024). A key symptom of and risk factor for depression is disturbed sleep; thus, improving mental health can result in improved sleep, and vice versa (Fang et al. 2019). PA increases brain‐derived neurotrophic factor (BDNF), which has been reported to alleviate depressive symptoms—illustrating a plausible neurophysiological pathway for PA to impact sleep (Uchida et al. 2012).

Most studies quantify objective sleep measures using gold‐standard polysomnography (PSG) or wearable biometric devices such as actigraphy wristwatches (Samson 2020). Self‐reported (or subjective) sleep quality is assessed through surveys and questionnaires, most commonly the Pittsburgh Sleep Quality Index or PSQI (Buysse et al. 1989). Similarly, objective PA is often measured via accelerometry (i.e., actigraphy is a form of accelerometry) and subjective PA is measured via surveys and questionnaires.

The relationship between PA and sleep has been the focus of many meta‐reviews (Kline et al. 2021; Kredlow et al. 2015; Kubitz et al. 1996; Wang and Boros 2021). Individuals who exercised regularly had longer objective total sleep time (TST), higher sleep efficiency (SE: percentage of time asleep out of total time spent in bed), shorter sleep latency (time it takes to fall asleep after lights out), and higher sleep quality than individuals who were not exercising regularly (Kredlow et al. 2015). Moderate‐intensity regular exercise was associated with improved subjective sleep quality (Kredlow et al. 2015; Wang and Boros 2021). It has been proposed that engaging in long durations of vigorous‐intensity PA may enhance the restorative functions of sleep (Brinkman, Reddy, and Sharma 2023; Dijk 2009), requiring longer and deeper sleep to repair cellular damage (e.g., muscle recovery and regeneration) that incurred during vigorous activities (Chennaoui et al. 2021; Dattilo et al. 2011). On the other hand, vigorous‐intensity PA may result in increased sleep disturbances (e.g., difficulty falling asleep, restlessness during sleep)—overtraining and overreaching in endurance athletes manifested in reductions in sleep duration and quality (Lastella et al. 2018). However, at this time, the effects of vigorous‐intensity PA on sleep duration and quality remain inconclusive (Stutz, Eiholzer, and Spengler 2019; Wang and Boros 2021). In summary, there is indisputable evidence for the positive effects of PA, especially moderate‐intensity PA, on sleep duration and quality (Kredlow et al. 2015; Wang and Boros 2021).

It is important to consider the effects of age and sex on PA and sleep separately. The negative association of age with PA has been well documented across global populations (Caldwell 2016; Sallis 2000; Troiano et al. 2008). There is mixed evidence for sleep duration and quality decreasing with age. TST and SE decreased while sleep latency and wake after sleep onset (WASO) increased with age among healthy adults in the Global North (Ohayon et al. 2004). Sleep problems were widely reported by older adults in low‐income settings across Asian and African countries (including rural populations) (Stranges et al. 2012). Conversely, Knutson (2014) did not find sleep duration and quality to decrease with age in a non‐electrified Haitian community, and Prall et al. (2018) found that older individuals had longer TST and higher SE in an agropastoral community in Namibia, likely due to age‐related labor demands. Sex and gender differences in sleep are commonly reported. Women typically had objectively better measures of sleep quality than men, but women tended to report subjectively poorer sleep—sex hormones and sex‐specific mechanisms in sleep regulation may underlie these differences (Mong and Cusmano 2016). With regard to sex differences in PA, most studies found PA to be higher in males than in females (Troiano et al. 2008) during adolescence and adulthood (Caldwell et al. 2023; Caspersen, Pereira, and Curran 2000) and among older adults (Lee 2005). It is important to note that differences between men and women in sleep and PA are strongly subject to cultural variations in sex‐ and gender‐specific labor demands and nighttime activities (Prall et al. 2018). Even after accounting for effects of age and sex, the overall positive effects of PA on sleep remain evident, pointing to the robustness of this association (Kline et al. 2021; Kredlow et al. 2015; Wang and Boros 2021).

The link between daytime PA and nighttime sleep has been well researched and well established. However, there remains a paucity of studies investigating the effects of PA on sleep in non‐urban and nonindustrial populations, particularly those whose daily routine involves labor‐intensive activities (i.e., lifestyle‐embedded activities rather than purposive exercise or leisure‐time PA). Individuals' baseline PA level may moderate the influence of PA on sleep (Kredlow et al. 2015). A review by Kubitz et al. (1996) found that the positive effects of chronic exercise (i.e., PA among athletes, highly fit individuals, and those undergoing training) on sleep were larger than that of acute exercise—maintaining high levels of PA yielded greater benefits for sleep. Additionally, considering that sleep loss can impede physical performance (Fullagar et al. 2015) and motor tasks such as balance (Umemura et al. 2022) and gait control (Umemura et al. 2021), poor nighttime sleep may affect activity levels the following day. The bidirectional effect of PA and sleep warrants further investigation (Chennaoui et al. 2015). In this study, we tested the hypothesis that in a highly active group, we would find a positive and bidirectional relationship between daytime PA and nighttime sleep. We examined how age, gender, and occupation influenced PA in a group of rural Basotho and Xhosa agropastoralists residing in the Eastern Cape province in South Africa. Older individuals were expected to engage in shorter moderate‐to‐vigorous‐intensity PA (MVPA) and longer sedentary activity. Herders, being more active while driving livestock, were anticipated to have longer MVPA and shorter sedentary activity durations than non‐herders, with no significant gender differences in activity levels once occupation was considered. We also analyzed the effects of daytime PA on sleep, predicting that longer durations of MVPA and low‐intensity activities would improve sleep quality and longer duration of MVPA might increase sleep duration. Additionally, we explored how previous night's sleep impacted daytime PA, expecting that insufficient sleep would lead to fatigue, reducing MVPA and increasing sedentary activity.

## 2 Materials and Methods

### 2.1 Ethics

This research has been approved by the study site's traditional leaders and the University of Toronto's Social Sciences, Humanities and Education Human Research Ethics Board (Protocol # 42393). After conveying the purpose and requirements of the study and translating contents of the consent form to Sesotho and Xhosa, participants gave their informed consent prior to participating in the study.

### 2.2 Study Site

We carried out this study in rural villages located in the Eastern Cape province in South Africa. Based on M. F. L.'s personal observations across three field seasons (totaling 9 months), and local authors' (P. L., T. P., T. L.) intimate knowledge of the region and community, we have gathered the following information on the study site and population. The villages fell along the Maloti‐Drakensberg Mountains, which ran across the South Africa–Lesotho border. This high‐altitude (~1550 m above sea level) and mountainous region consisted of a mosaic of residential households, small private crop fields, large community fields, grasslands (pasture), and forest fragments. At the study site, six main villages made up the traditional jurisdiction of the principal chief, or chiefess. Each main village was governed by a hereditary headman, or chief (*morena*), and several elected subheadmen. We primarily worked in two main villages, which were governed by two separate chiefs. Main village jurisdictions also encompassed smaller adjacent villages that ranged from 25 to over 2000 inhabitants (*personal observations*).

The main ethnic groups in this community were the Basotho and Xhosa, whose main languages were Sesotho and Xhosa, respectively. While there were cultural differences between the Basotho and Xhosa, they did not differ in subsistence strategy or sleep practices. Most villages were a mix of different ethnic groups, and inter‐ethnic marriages and cohabitation (i.e., herders that room together) were common. Villagers typically practiced subsistence and small‐scale cash‐crop agriculture using the available land on their allotted 50‐m by 50‐m household property. Common crops grown included maize, carrot, pumpkin, turnip, cabbage, spinach, beans, potato, and fruit trees. Agriculture (farming) was often coupled with pastoralism (herding) and most adults residing in the village owned and/or herded livestock as part of their daily routine. Common larger livestock were cattle, sheep, and goats, and smaller livestock were pigs, chickens, and ducks. Participants obtained their foods from grocery shops in the nearby town, smaller local convenience shops (*spaza*), and locally grown vegetables. Meals almost always included the staple starch maize meal (*pap*). Many households had horses that provided crucial means of transportation for men in this remote region and donkeys that helped carry heavy items across difficult terrain. Agropastoralism was the main subsistence strategy in this region, and very few people received a steady wage‐based income. This meant that farming and herding made up a significant portion of villagers' daily activity. Sources of monetary income included selling livestock and livestock products, selling firewood, selling homemade beer, bricklaying, farm labor, running spaza shops, and receipt of government grants (e.g., pension, child support grant).

### 2.3 Participant Recruitment

We used door‐to‐door recruitment based on authors' knowledge of the village population and the aims of this project. Our recruitment was purposive, with clearly defined sample sizes based on a priori power analysis, and we strived for even sampling across occupation groups. We prioritized participants who maintained a consistent occupation during the data collection period. We tried to space out households across villages; however, our recruitment was not random. We approached households based on whether they had livestock or not (i.e., herders versus non‐herders), and this was based on local coauthors' (P. L., T. P., T. L.) knowledge of the community. For cattle post herders, we started by visiting all cattle posts that were accessible by foot and within a 3‐h hike from the villages and used snowball sampling until our desired sample size was reached. After the first field season, we targeted suitable returning participants in subsequent seasons and recruited new participants as needed to meet our desired sample size. During recruitment, we introduced ourselves, explained our project, listed what will be asked of participants if they agree to participate, and read the contents of the consent form. The authors P. L., T. P., and T. L., who are fluent in Sesotho and Xhosa, translated our conversations.

### 2.4 Data Collection

We collected data across three field seasons, from April 27 to July 1, 2022, January 25 to April 13, 2023, and May 30 to August 3, 2023. We recruited 73 participants (20 women, 53 men) during season 1, 83 (24 women, 59 men) during season 2, and 89 (22 women, 67 men) during season 3—there were numerous returning participants across seasons. Participants were between ages 14 and 85 (mean: 41.6 years) as this included younger herd boys and older herdsmen. The sample size was not gender‐balanced because the larger project focused on herders, which were men in this community. Seasons were divided as follows: summer (January 1 to March 31), fall (April 1 to May 31), and winter (June 1 to August 31).

We recruited a total of 231 participants across three field seasons (some were returning participants, so the number of unique participants was 135 for a total of 7125 individual days). We excluded individuals that had less than 5 days of complete actigraphy data; thus, we report results from 228 total participants (133 unique participants; 7111 individual days).

During each field season, we assigned participants' primary occupation as either (1) non‐herder (NH): individuals who do not take care of livestock during the day, which included all the women; (2) village herder (VH): individuals residing in more densely populated villages who take care of livestock (i.e., take them to the pasture) during the day; or (3) cattle post herder (CPH): individuals residing in remote cattle posts in the mountains who take care of livestock during the day. The field research team (M. F. L., P. L., T. P.) determined participants' occupation during the recruitment process (i.e., we explicitly asked if they owned or took care of livestock and what their main tasks were during the day). For instance, some men self‐identified as herders because they owned livestock; however, they employed other herders to take care of the animals during the day; thus, we classified these men as non‐herders in our study. In the rare cases when a participant's occupation changed during the study (e.g., a non‐herder helped take care of his neighbors' animals for a few days), we assigned their primary occupation as the one they held for the majority (> 50%) of the study period. We aimed for even sampling across women NH, men NH, men VH, and men CPH. We did not specify whether participants were farmers since the majority of households grew crops but farming labor demands varied greatly (i.e., some tended to fields occasionally, while others spent all day in the fields).

We used CamNTech's MotionWatch 8 and MotionWatch Rugged to quantify participants' daily activity and sleep measures. Actigraphy devices provide a non‐invasive method to study activity and sleep outside of laboratory and clinical settings (Samson 2020). The actigraphy wristwatch detects movement and acceleration and records them as activity counts for a pre‐defined time epoch. These activity counts are then used to calculate different PA levels and sleep measures based on cut‐off points (Landry et al. 2015). We asked all participants to wear the wristwatch on their non‐dominant wrist for at least 1 month during each field season. We set all MotionWatch to uniaxial mode to detect movement along the *z*‐axis—this recording mode has been validated against polysomnography for generating accurate sleep measures (de Souza et al. 2003). We set the sampling frequency to 1 min to generate activity counts per minute (0.0167 Hz). We also collected demographic information on participants (age, gender, occupation status, farming, and livestock responsibilities).

### 2.5 Data Processing and Analyses

We imported raw actigraphy data into MotionWare (v1.3.33) and manually cleaned the data by omitting non‐wear times that exceeded 30 min. If any non‐wear time occurred during a sleep period, we excluded the entire sleep period. We visually scored sleep periods by selecting the period between when participants went to bed (i.e., “lights out,” which was characterized by a notable decrease in activity) and “got up” (characterized by a marked and steady increase in activity). Using an epoch‐by‐epoch designation of sleep/awake states, MotionWare algorithmically determined the fell asleep time (“sleep onset”), which occurred after lights out, and woke up time (“sleep offset”), which occurred before got up. See Appendix A and Figure A1 for more details on visual scoring of sleep periods in MotionWare. After setting the threshold to the validated “high” sensitivity setting, the “sleep analysis” function generated a list of sleep measures for each identified sleep period. In this study, we looked at total sleep time (TST), sleep efficiency (SE), fragmentation index (FI), and percentage wake after sleep onset (% WASO). TST is the total time spent in sleep during the identified sleep period. SE is the total sleep time as a percentage of time in bed. FI is an indication of how fragmented the sleep period was and is the sum of the percentage mobile time and immobile bouts less than or equal to 1 min. Percentage (%) WASO is the total time spent in wake expressed as a percentage of the assumed sleep time.

After excluding all sleep periods and non‐wear times, we used the “physical activity analysis” function to generate a summary of daily durations of different intensities of PA for each participant. In this study, we looked at durations of the following activity intensities: total vigorous‐intensity PA or VPA (> 1000 counts per minute, or cpm), total moderate‐intensity PA or MPA (500–999 cpm), total low‐intensity PA or LPA (50–499 cpm), total sedentary activity (1–49 cpm), bouts of VPA, and bouts of MPA. For bouts of VPA and bouts of MPA, the duration of the activity had to be 10 min or more. Moderate‐to‐vigorous‐intensity PA, or MVPA, was calculated as the summation of VPA and MPA. The default thresholds that MotionWare used were determined empirically by having a small group of healthy adults do a 6‐minute walk test where participants were asked to perform activities at different intensities based on the 2013 United Kingdom's National Institute for Health and Care Excellence (NICE) guidelines (MotionWatch & MotionWare User Guide: Issue 1.3.17a, pp. 63). These cut‐off points were then averaged and rounded to give the default values of 50/500/100. Similar methods for establishing cut‐off points for actigraphy devices can be found in Hickey et al. (2016). We did not perform individual‐calibration test, which we discuss in the Limitations section. See Appendix A, Table A1 for definitions of all sleep and activity measures.

We first built three linear mixed‐effects models (LMMs) with the following daily PA measures as the response variable (i.e., each row in our dataset was 1 day for one individual): % total MVPA, % total LPA, and % total sedentary activity. We used the percentage of each activity duration out of daily wear time duration to account for different wear times. We included gender (binary: men, women (reference group)), age (continuous), and occupation (categorical: non‐herder (reference group), cattle post herder, village herder) as the main predictor variables. Based on previous studies on other subsistence groups (e.g., Yetish et al. 2015), we know that seasonal differences in environmental conditions and labor demands will likely lead to seasonal differences in PA and sleep; thus, we included season (categorical: summer (reference group), fall, winter) as a covariate in our models. We set subject ID as a random effect to account for repeated observations.

To assess which measures of daytime PA best accounted for variation in nighttime sleep, we built four LMMs with TST, SE, FI, and % WASO as response variables. For daytime activity measures, we included total MVPA, bouts of MVPA, and total LPA durations. We used the duration of PA activities since we wanted to evaluate whether the amount of time spent in different PA intensities would affect sleep measures. We included season, gender, age, and occupation as covariates and set subject ID as a random effect. We first used the model selection tool “dredge” to generate candidate models and then averaged models with ∆AIC_C_ < 10. We report the full average coefficients and standard errors, as it decreases the effect sizes and errors of predictor variables with weak effects on the response variable (Grueber et al. 2011).

To assess how daytime PA was affected by the previous night's sleep measures, we constructed three LMMs with % total MVPA, % total LPA, and % total sedentary activity durations as the response variables. We first included the previous night's TST, SE, FI, and % WASO as the main predictors and included season, gender, age, and occupation as covariates and set subject ID as a random effect. Variance inflation factor (VIF) indicated moderate correlation between previous night's SE and % WASO; thus, to avoid multicollinearity, we removed SE since we were more interested in whether nighttime awakenings affected next day's PA. We used the same model averaging protocol as mentioned above.

Finally, to tease apart the effects of gender and occupation on daytime PA (% total MVPA, % total LPA, % total sedentary activity) and nighttime sleep (TST, SE, FI, % WASO), we constructed three additional LMMs for each variable. Model 1 only included gender as a predictor and omitted occupation. Model 2 included gender as a predictor but only included non‐herders, since only non‐herders consisted of both men and women. Model 3 included occupation as a predictor but only included men, since only men had more than one occupation type. All models included age and season as covariates and set subject ID as a random effect.

All statistical analyses were performed in R Studio (v.2023.12.1+402; R v.4.0.1) for Mac OS X (R Core Team 2020). We used the following packages: “lme4” for fitting LMMs (Bates et al. 2015), “Car” (Fox and Weisberg 2019) and “DHARMa” for model diagnostics (Hartig 2020), and MuMIn for model averaging (Bartoń 2020). We used “dotwhisker” (Solt and Hu 2021), “egg” (Auguie 2019), and “ggplot2” (Wickham 2016) for creating figures. We looked at the VIF for all models and confirmed low correlation for all predictors (VIF < 5). Overall model significance was determined by comparing the final model with the intercept‐only model using a likelihood ratio test. All statistical tests were two‐tailed, with alpha set to 0.05 for significance.

## 3 Results

**FIGURE 1:**
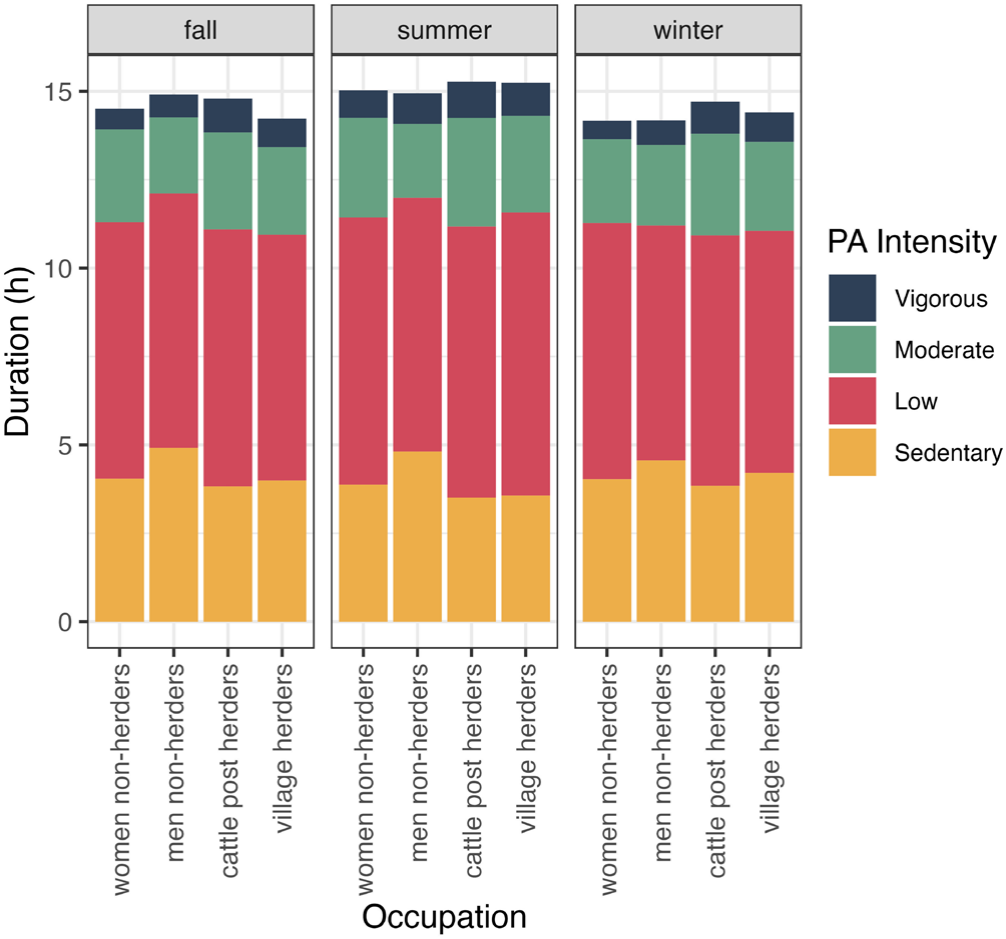
Stacked bar plot of the mean daily duration of vigorous (navy), moderate (green), low (red), and sedentary (yellow) physical activities separated by season and occupation: women non‐herders, men non‐herders, cattle post herders (all men), and village herders (all men).

Descriptive statistics for daytime PA and nighttime sleep measures are reported in Table 1. The mean duration of data collection per participant was 31.19 days (±9.56, range: 5–61 days). The mean TST was 6.95 h (±0.97), SE was 74.35% (±6.88), FI was 41.14 (±9.20), and % WASO was 21.68% (± 6.20). We found that individuals spent a mean of 201.8 min per day (±79.4) in total MVPA—most of which consisted of moderate‐intensity activity, 425.8 min per day (±62.7) in total LPA, and 247.3 min per day (±77.3) in sedentary activity (Figure 1).

**TABLE 1:** Descriptive statistics (mean and standard deviation [SD]) for daytime physical activity and nighttime sleep measures, grouped by gender and occupation.

	Overall	Women	Men		
	(*n* = 133)	NH (*n* = 88)	NH (*n* = 57)	VH (*n* = 102)	CPH (*n* = 85)
Age (years)	41.6 (16.1)	49.0 (14.0)	43.3 (17.2)	40.5 (16.0)	35.71 (13.6)
Wear time (min)	874.78 (53.33)	870.87 (56.34)	870.58 (60.18)	859.60 (67.79_	880.83 (70.53)
Mean (SD) of daytime physical activity durations (min)					
Total VPA	47.18 (30.83)	34.73 (30.29)	43.57 (28.15)	49.74 (32.84)	56.54 (30.15)
Total MPA	154.57 (54.83)	149.38 (60.62)	129.25 (57.61)	150.57 (47.59)	172.64 (49.62)
Total MVPA	201.75 (79.43)	184.11 (86.76)	172.82 (78.06)	200.31 (72.89)	229.19 (72.15)
Bouts of VPA	6.77 (7.35)	3.31 (6.15)	7.70 (8.60)	8.79 (9.69)	7.84 (11.07)
Bouts of MPA	66.38 (40.99)	50.13 (43.87)	57.31 (38.54)	67.24 (41.07)	79.34 (41.12)
Bouts of MVPA	73.14 (46.41)	53.44 (49.19)	65.01 (44.25)	76.03 (48.68)	87.18 (47.46)
Total LPA	425.75 (62.68)	447.05 (79.49)	416.87 (62.08)	427.07 (70.40)	428.94 (59.31)
Total sedentary	247.28 (71.18)	239.71 (77.32)	280.89 (91.00)	242.22 (63.63)	222.70 (55.38)
Mean (SD) of nighttime sleep measures					
TST (h)	6.95 (0.97)	7.18 (0.89)	6.78 (0.94)	6.95 (0.98)	6.70 (1.01)
SE (%)	74.35 (6.88)	76.32 (6.17)	72.71 (6.41)	73.87 (5.98)	73.87 (7.83)
FI	41.14 (9.20)	37.77 (9.40)	42.57 (9.41)	42.96 (9.20)	41.10 (9.72)
WASO (h)	1.94 (0.53)	1.78 (0.51)	2.12 (0.64)	2.03 (0.54)	1.90 (0.64)
% WASO	21.68 (6.20)	19.74 (5.54)	23.52 (6.30)	22.36 (5.46)	21.89 (7.32)

Abbreviations: CPH, cattle post herder; FI, fragmentation index; LPA, low‐intensity physical activity; MPA, moderate‐intensity physical activity; MVPA, moderate‐to‐vigorous‐intensity physical activity; NH, non‐herder; SE, sleep efficiency; TST, total sleep time; VH, village herder; VPA, vigorous‐intensity physical activity; WASO, wake after sleep onset.

### 3.1 Gender, Age, and Occupation‐Level Differences in Daytime PA

**FIGURE 2:**
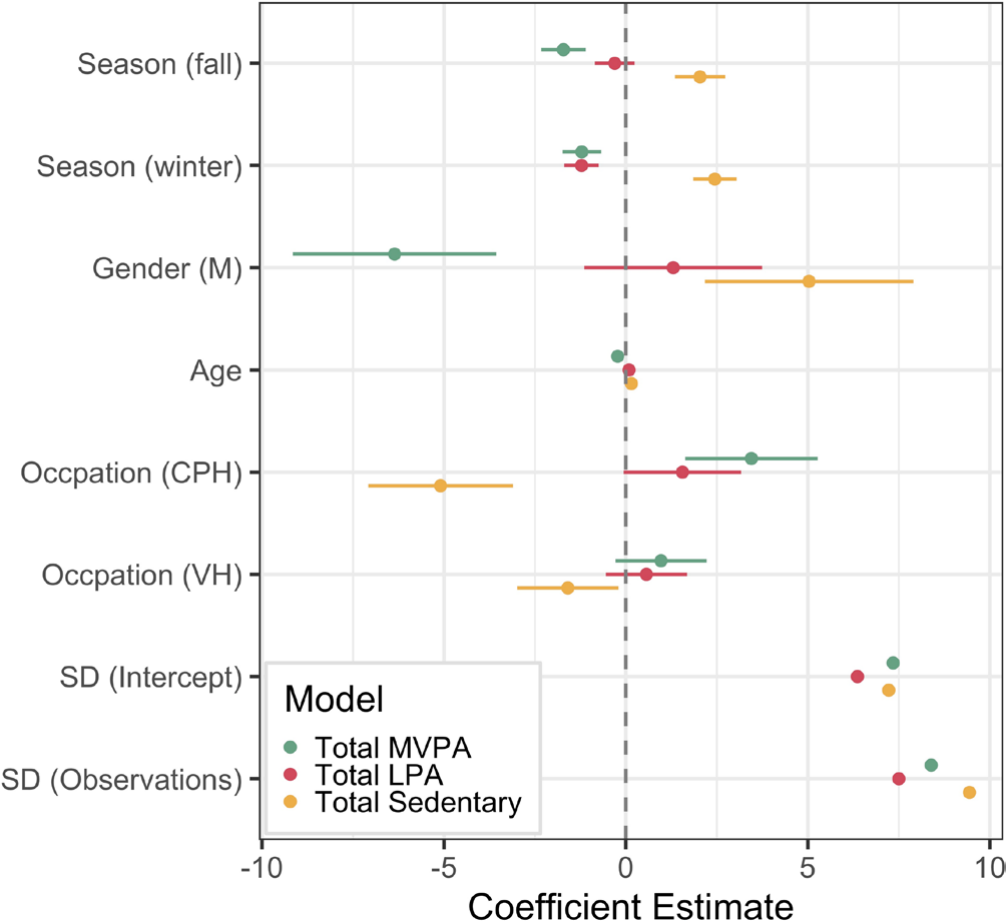
Coefficient plot of linear mixed‐effects models for the effects of season, gender, age, and occupation on daytime total MVPA (green), total LPA (red), and total sedentary (yellow) activity durations. The reference group for season was summer, for gender was women, and for occupation was non‐herders. CPH, cattle post herder; LPA, low‐intensity physical activity; MVPA, moderate‐to‐vigorous‐intensity physical activity; NH, non‐herder; VH, village herder.

In the daytime PA models, we found gender, age, and occupation differences (Table 2; Figure 2). Compared to women, men had significantly lower % total MVPA (*β* = −6.36, *p* < 0.001, 95% CI [−9.19, −3.52]) and longer % total sedentary activity (*β* = 5.03, *p* < 0.001, 95% CI [2.18, 7.89]). Older people had lower % total MVPA (*β* = −0.228, *p* < 0.001, 95% CI [−0.307, −0.147]), higher % total LPA activities (*β* = 0.086, *p* = 0.014, 95% CI [0.017, 0.154]), and higher % total sedentary activity (*β* = 0.150, *p* < 0.001, 95% CI [0.072, 0.228]) compared to younger people. There was no effect of gender on % total LPA duration. We added an interaction term between gender and age, but since they were not significant in any of the models, we excluded them from the reported models. Among the three occupation groups, we found that cattle post herders (CPHs) had significantly greater % total MVPA duration than non‐herders (*β* = 3.45, *p* < 0.001, 95% CI [1.64. 5.27]), and both cattle post and village herders (VHs) had lower % sedentary activity (CPH: *β* = −5.09, *p* < 0.001, 95% CI [−7.08, −3.12]; VH: *β* = −1.60, *p* = 0.025, 95% CI [−3.00, −0.212]) than non‐herders. There was no significant occupation effect on % total LPA.

**TABLE 2:** Summary of LMMs on the effects of season, gender, age, and occupation on daytime physical activity measures. Significant effects (*p* < 0.05) are shown in bold.

Variable	*β* (SE)	95% CI (low, high)	*p*
% Total MVPA (overall model*: N* = 7111, *χ* ^2^ = 73.5, *p* < 0.001)			
Intercept	36.91 (2.43)	32.14, 41.67	< 0.001
Season (ref: summer)	—	—	—
**Fall**	**−1.72 (0.314)**	**−2.33, −1.10**	**< 0.001**
**Winter**	**−1.21 (0.271)**	**−1.75, −0.683**	**< 0.001**
Gender (ref: women)	—	—	—
**Men**	**−6.36 (1.43)**	**−9.19, −3.52**	**< 0.001**
**Age**	**−0.228 (0.040)**	**−0.307, −0.147**	**< 0.001**
Occupation (ref: NH)	—	—	—
**CPH**	**3.45 (0.927)**	**1.64. 5.27**	**< 0.001**
VH	0.964 (0.640)	−0.288, 2.22	0.132
% Total LPA (overall model: *N* = 7111, *χ* ^2^ = 40.2, *p* < 0.001)			
Intercept	44.36 (2.11)	40.23, 48.48	< 0.001
Season (ref: summer)	—	—	—
Fall	−0.208 (0.280)	−0.856, 0.242	0.272
**Winter**	**−1.22 (0.242)**	**−1.69, −0.745**	**< 0.001**
Gender (ref: women)	—	—	—
Men	1.30 (1.25)	−1.24, 3.83	0.299
**Age**	**0.086 (0.035)**	**0.017, 0.154**	**0.014**
Occupation (ref: NH)	—	—	—
CPH	1.55 (0.824)	−0.053, 3.170	0.059
VH	0.564 (0.570)	−0.549, 1.69	0.323
% Total sedentary (overall model: *N* = 7111, *χ* ^2^ = 110, *p* < 0.001)			
Intercept	18.41 (2.42)	13.68, 23.16	< 0.001
Season (ref: summer)	—	—	—
**Fall**	**2.04 (0.352)**	**1.35, 2.73**	**< 0.001**
**Winter**	**2.44 (0.304)**	**1.85, 3.04**	**< 0.001**
Gender (ref: women)	—	—	—
**Men**	**5.03 (1.46)**	**2.18, 7.89**	**< 0.001**
**Age**	**0.150 (0.040)**	**0.072, 0.228**	**< 0.001**
Occupation (ref: NH)	—	—	—
**CPH**	**−5.09 (1.01)**	**−7.08, −3.12**	**< 0.001**
**VH**	**−1.60 (0.710)**	**−3.00, −0.212**	**0.025**

Abbreviations: CPH is cattle post herder; LPA, low‐intensity physical activity; MVPA, moderate‐to‐vigorous‐intensity physical activity; NH, non‐herder; VH, village herder.

There were significant seasonal differences in all daytime PA measures. Compared to summer, % total MVPA was lower in both the fall (*β* = −1.72, *p* < 0.001, 95% CI [−2.33, −1.10]) and winter (*β* = −1.21, *p* < 0.001, 95% CI [−1.75, −0.683]), % total LPA was lower in the winter (*β* = −1.22, *p* < 0.001, 95% CI [−1.69, −0.745]), and % total sedentary activity was higher in both the fall (*β* = 2.04, *p* < 0.001, 95% CI [1.35, 2.73]) and winter (*β* = 2.44, *p* < 0.001, 95% CI [1.85, 3.04]).

### 3.2 Effects of Daytime PA on Nighttime Sleep

In the second set of averaged models looking at how daytime activity affected nighttime sleep (Table 3), we found that TST was negatively associated with total MVPA (*β* = −0.118, *p* < 0.001, 95% CI [−0.162, −0.073]), bouts of MVPA (*β* = −0.105, *p* < 0.001, 95% CI [−0.144, −0.066]), and total LPA durations (*β* = −0.364, *p* < 0.001, 95% CI [−0.389, −0.339]). SE increased with total LPA duration (*β* = 0.104, *p* < 0.001, 95% CI [0.080, 0.127]). FI was negatively affected by total MVPA (*β* = −0.049, *p* = 0.019, 95% CI [−0.091, −0.008]). Both FI (*β* = −0.138, *p* < 0.001, 95% CI [−0.163, −0.113]) and % WASO (*β* = −0.151, *p* < 0.001, 95% CI [−0.174, −0.128]) were negatively affected by total LPA durations.

**TABLE 3:** Summary of full model‐averaged coefficients from LMMs on the effects of daytime physical activities on nighttime sleep measures. Significant effects (*p* < 0.05) are shown in bold.

Variable	*β* (SE)	95% CI (low, high)	*p*
TST			
**Total MVPA**	**−0.118 (0.023)**	**−0.162, −0.073**	**< 0.001**
**Bouts of MVPA**	**−0.105 (0.020)**	**−0.144, −0.066**	**< 0.001**
**LPA**	**−0.364 (0.013)**	**−0.389, −0.339**	**< 0.001**
Season (ref: summer)	—	—	—
**Fall**	**0.058 (0.013)**	**0.033, 0.083**	**< 0.001**
**Winter**	**0.123 (0.013)**	**0.100, 0.151**	**< 0.001**
Gender (ref: women)	—	—	—
**Men**	**−0.166 (0.045)**	**−0.254, −0.078**	**< 0.001**
Age	0.007 (0.029)	−0.049, 0.063	0.806
Occupation (ref: NH)	—	—	—
CPH	−0.012 (0.028)	−0.068, 0.044	0.670
VH	0.036 (0.036)	−0.035, 0.107	0.325
SE			
Total MVPA	0.001 (0.008)	−0.014, 0.016	0.854
Bouts of MVPA	−0.001 (0.007)	−0.014, 0.012	0.862
**Total LPA**	**0.104 (0.012)**	**0.080, 0.127**	**< 0.001**
Season (ref: summer)	—	—	—
**Fall**	**0.034 (0.013)**	**0.009, 0.058**	**0.007**
**Winter**	**0.070 (0.013)**	**0.045, 0.095**	**< 0.001**
Gender (ref: women)	—	—	—
**Men**	**−0.193 (0.055)**	**−0.302, −0.084**	**< 0.001**
Age	0.006 (0.027)	−0.047, 0.059	0.821
Occupation (ref: NH)	—	—	—
CPH	−0.025 (0.038)	−0.099, 0.049	0.504
**VH**	**0.103 (0.028)**	**0.048, 0.158**	**< 0.001**
FI			
**Total MVPA**	**−0.049 (0.021)**	**−0.091, −0.008**	**0.019**
Bouts of MVPA	0.002 (0.013)	−0.024, 0.029	0.858
**Total LPA**	**−0.138 (0.013)**	**−0.163, −0.113**	**< 0.001**
Season (ref: summer)	—	—	—
Fall	−0.002 (0.007)	−0.015, 0.012	0.794
Winter	−0.001 (0.006)	−0.013, 0.011	0.886
Gender (ref: women)	—	—	—
**Men**	**0.167 (0.054)**	**0.062, 0.273**	**0.002**
Age	0.019 (0.045)	−0.069, 0.106	0.674
Occupation (ref: NH)	—	—	—
CPH	−0.001 (0.038)	−0.075, 0.073	0.976
VH	−0.057 (0.031)	−0.118, 0.005	0.070
% WASO			
Total MVPA	−0.015 (0.019)	−0.051, 0.022	0.428
Bouts of MVPA	−0.002 (0.009)	−0.019, 0.015	0.846
**Total LPA**	**−0.151 (0.012)**	**−0.174, −0.128**	**< 0.001**
Season (ref: summer)	—	—	—
**Fall**	**−0.050 (0.012)**	**−0.074, −0.026**	**< 0.001**
**Winter**	**−0.080 (0.013)**	**−0.105, −0.055**	**< 0.001**
Gender (ref: women)	—	—	—
**Men**	**0.205 (0.052)**	**0.102, 0.308**	**< 0.001**
Age	−0.003 (0.018)	−0.038, 0.033	0.873
Occupation (ref: NH)	—	—	—
CPH	0.001 (0.036)	−0.069, 0.072	0.971
**VH**	**−0.065 (0.029)**	**−0.123, −0.008**	**0.026**

Abbreviations: CPH, cattle post herder; FI, fragmentation index; LPA, low‐intensity physical activity; MVPA, moderate‐to‐vigorous‐intensity physical activity; NH, non‐herder; SE, sleep efficiency; TST, total sleep time; VH, village herder; WASO, wake after sleep onset.

Compared to summer, TST was significantly longer and SE was higher in the fall (TST: *β* = 0.058, *p* < 0.001, 95% CI [0.033, 0.083]); SE: (*β* = 0.034, *p* = 0.007, 95% CI [0.009, 0.058]) and winter (TST: *β* = 0.123, *p* < 0.001, 95% CI [0.100, 0.151]); SE: (*β* = 0.070, *p* < 0.001, 95% CI [0.045, 0.095]). Compared to summer, percentage WASO was lower in the fall (*β* = −0.050, *p* < 0.001, 95% CI [−0.074, −0.026]) and winter (*β* = −0.080, *p* < 0.001, 95% CI [−0.105, −0.055]). There was no seasonal difference in FI. Compared to women, men had shorter TST (*β* = −0.166, *p* < 0.001, 95% CI [−0.254, −0.078]), lower SE (*β* = −0.193, *p* < 0.001, 95% CI [−0.302, −0.084]), higher FI (*β* = 0.167, *p* = 0.002, 95% CI [0.062, 0.273]), and higher % WASO (*β* = 0.205, *p* < 0.001, 95% CI [0.102, 0.308]). Age did not have an effect on any sleep measures, and the only occupation difference we found was that village herders had higher SE (*β* = 0.103, *p* < 0.001, 95% CI [0.048, 0.158]) and lower % WASO (*β* = −0.065, *p* = 0.026, 95% CI [−0.123, −0.008]) than non‐herders.

### 3.3 Effects of Nighttime Sleep Measures on Daytime PA

Results from models looking at the effects of previous night's sleep measures on daytime PA showed that none of the sleep measures (TST, FI, % WASO) had significant effects on any of the daytime PA measures (% total MVPA, % total LPA, % total sedentary activity). Full model results can be found in Table 4.

**TABLE 4:** Summary of full model‐averaged coefficients from LMMs on the effects of previous night's sleep measures on daytime physical activities. Significant effects (*p* < 0.05) are shown in bold.

Variable	*β* (SE)	95% CI (low, high)	*p*
% Total MVPA			
Prev. TST	0.002 (0.005)	−0.009, 0.012	0.780
Prev. FI	0.00001 (0.001)	−0.002, 0.002	0.986
Prev. % WASO	0.001 (0.005)	−0.009, 0.011	0.842
Season (ref: summer)	—	—	—
**Fall**	**−0.059 (0.011)**	**−0.081, −0.037**	**< 0.001**
**Winter**	**−0.058 (0.012)**	**−0.081, −0.035**	**< 0.001**
Gender (ref: women)	—	—	—
**Men**	**−0.205 (0.054)**	**−0.311, −0.098**	**< 0.001**
**Age**	**−0.327 (0.054)**	**−0.433, −0.221**	**< 0.001**
Occupation (ref: NH)	—	—	—
**CPH**	**0.118 (0.034)**	**0.051, 0.186**	**< 0.001**
VH	0.024 (0.025)	−0.025, 0.074	0.340
% Total LPA			
Prev. TST	−0.016 (0.017)	−0.049, 0.017	0.343
Prev. FI	−0.002 (0.008)	−0.018, 0.013	0.792
Prev. % WASO	−0.007 (0.014)	−0.034, 0.021	0.642
Season (ref: summer)	—	—	—
Fall	−0.011 (0.013)	−0.035, 0.014	0.385
**Winter**	**−0.056 (0.013)**	**−0.0817, −0.030**	**< 0.001**
Gender (ref: women)	—	—	—
Men	0.010 (0.047)	−0.081, 0.102	0.826
Age	0.071 (0.085)	−0.096, 0.238	0.405
Occupation (ref: NH)	—	—	—
CPH	0.048 (0.045)	−0.041, 0.136	0.290
VH	0.018 (0.026)	−0.032, 0.068	0.488
% Total sedentary			
Prev. TST	0.009 (0.011)	−0.008, 0.009	0.420
Prev. FI	0.016 (0.011)	−0.005, 0.005	0.158
Prev. % WASO	0.001 (0.012)	−0.003, 0.003	0.908
Season (ref: summer)	—	—	—
**Fall**	**0.066 (0.012)**	**0.041, 0.090**	**< 0.001**
**Winter**	**0.102 (0.013)**	**0.077, 0.127**	**< 0.001**
Gender (ref: women)	—	—	—
**Men**	**0.169 (0.056)**	**0.060, 0.278**	**< 0.001**
**Age**	**0.203 (0.053)**	**0.079, 0.317**	**< 0.001**
Occupation (ref: NH)	—	—	—
**CPH**	**−0.180 (0.037)**	**−0.252, −0.109**	**< 0.001**
VH	−0.051 (0.027)	−0.104, 0.003	0.064

Abbreviations: CPH, cattle post herder; FI, fragmentation index; LPA, low‐intensity physical activity; MVPA, moderate‐to‐vigorous‐intensity physical activity; NH, non‐herder; TST, total sleep time; VH, village herder; WASO, wake after sleep onset.

### 3.4 Additional Analyses on Effects of Gender and Occupation on Daytime PA and Nighttime Sleep

When excluding occupation as a predictor in the model (Model 1), we found that men had significantly lower % total MVPA (*β* = −5.34, *p* < 0.001) and higher % total sedentary activity (*β* = 3.20, *p* = 0.024) than women. When only including non‐herders (Model 2), we found that men had significantly longer % total sedentary activity than women (*β* = 6.01, *p* = 0.013). When only including men (Model 3), we found that cattle post and village herders had significantly higher % total MVPA (CPH: *β* = 3.19, *p* < 0.001; VH: *β* = 1.30, *p* = 0.046) and lower % total sedentary activity (CPH: *β* = −5.13, *p* < 0.001; VH: *β* = −1.65, *p* = 0.023) than non‐herders, and cattle post herders had higher % total LPA than non‐herders (*β* = 1.98, *p* = 0.012).

When excluding occupation as a predictor in the model (Model 1), we found that men had significantly shorter TST (*β* = −0.450, *p* = 0.007), higher FI (*β* = 5.63, *p* < 0.001), and higher % WASO (*β* = 2.58, *p* = 0.017) than women. When only including non‐herders (Model 2), we found that men had significantly shorter TST (*β* = −0.461, *p* = 0.032), lower SE (*β* = −4.35, *p* = 0.005), higher FI (*β* = 7.13, *p* = 0.003), and higher % WASO (*β* = 4.29, *p* = 0.003) than women. When only including men (Model 3), we found that cattle post herders had significantly shorter TST than non‐herders (*β* = −0.327, *p* = 0.023) and village herders had significantly higher SE (*β* = 2.32, *p* < 0.001), lower FI (*β* = −2.13, *p* = 0.033), and lower % WASO (*β* = −1.58, *p* = 0.006) than non‐herders. Results from these models can be found in Table S1.

## 4 Discussion

Our hypothesis was partially supported as we found positive, but not reciprocal, relationships between daytime PA and nighttime sleep quality among active rural agropastoralists. To our knowledge, this is the first study to objectively assess the association between daytime PA and sleep in a habitually active subsistence community (see Yetish, Kaplan, and Gurven (2018) for association between nighttime activities and sleep). We found that older people and men spent less percentage of their day in total MVPA and more percentage in sedentary activity compared to younger people and women, respectively. Among occupation types, we found that herders had a lower daily percentage of total sedentary activity than non‐herders, and cattle post herders had longer percentage total MVPA than non‐herders. When we examined the effects of daytime PA on nighttime sleep, we found that total MVPA and especially total LPA durations positively affected sleep quality (increased SE, decreased FI, and WASO). Conversely, TST was negatively impacted by total MVPA, bouts of MVPA, and LPA durations. We did not find evidence of previous night's sleep having any effects on daytime PA. In summary, there was significant age, gender, and occupation‐level differences in the duration and intensity of daytime PA. Additionally, our results point to a unidirectional relationship between PA and sleep where daytime PA affected nighttime sleep—the nature of this relationship differed for sleep duration versus quality.

### 4.1 Age‐Related Decrease in PA but Not Sleep

We found a decrease in % total MVPA and an increase in sedentary activity with age (although effect sizes were small; Figure 2), supporting the consensus that PA tends to decrease as we get older (Caspersen, Pereira, and Curran 2000; Sallis 2000; Troiano et al. 2008). Whether this age‐related difference in PA was primarily driven by biological or cultural influences remains unclear; likely a combination of both. The cultural norm in this community was to delegate labor‐intensive tasks (e.g., plowing fields, herding livestock) to younger members of the household or youth in the community (e.g., hired farmhands and herders), which could explain why durations of higher‐intensity PA decreased with age. Notably, % total LPA tended to increase among older participants, indicating that older members in this community were remaining active throughout the day, but they were allocating more time to low‐intensity activities. Among both Hadza hunter‐gatherers and Pokot pastoralists, PA decreased with age, but older participants were still highly active compared to older adults in the United States (Sayre, Pike, and Raichlen 2019; Sayre et al. 2020, 2023). Rural populations tend to spend less time in sedentary and more time in PA than those living in urban areas (Assah et al. 2015; Castrillon et al. 2010; Jayamani et al. 2013; Pratt et al. 2020). Older members of this community maintained daily PA levels by engaging in low‐intensity activities such as walking (i.e., due to remoteness of rural villages and lack of personal vehicles) and tending to gardens and crops.

Age had neither a negative or positive effect on sleep duration and quality. Our findings are in line with results from studies in nonindustrial groups (e.g., Knutson 2014; Prall et al. 2018) but counter to results derived from studies mostly conducted in the Global North (Espiritu 2008; Ohayon et al. 2004). Hood, Bruck, and Kennedy (2004) found that sleep quality among aged adults (65–85 years) was positively influenced by daily PA and exposure to ambient light (i.e., strengthened entrainment of the sleep–wake pattern to the light–dark cycle). Indeed, villagers in this rural community spent a considerable portion of their day exposed to natural light, which was in large part due to limited access to electricity and electrical devices (e.g., cell phones, television, artificial light). As mentioned, we did not find low activity durations to decrease with age, which was encouraging since low activity was found to have the most consistent positive effects on sleep quality (increased SE, decreased FI, decreased WASO). Building on Knutson's (2014) conclusion that sleep does not inevitably get worse as we get older, the widely accepted and cited age‐related decline in sleep may be attributed to extrinsic factors such as the degree of industrialization and electrification, exposure to the outdoors, and cultural contexts for daytime activities and nighttime sleep practices. We should point out that while the upper age range of study participants was 85, only ~10% were 65 years or older; therefore, we may not have adequately captured sleep changes among older adults.

### 4.2 Gender and Occupation‐Level Differences in Daytime and Nighttime Activities

In agropastoral societies where the division of labor is more flexible and evenly distributed, there tends to be less discrepancy in PA between men and women (Kashiwazaki et al. 2009); this was not the case in this study group. Although a majority of subsistence populations studied to date showed higher PA in men compared to women, several studies found the opposite (Gurven et al. 2013, Table 2). We found that men spent more percentage of their day in sedentary activity compared to women—this was true among all occupations and among non‐herder men and women. We also found that men spent less percentage of their day in MVPA than women, which was the same finding as Sarma et al.'s (2020) study on BaYaka foragers. Conversely, among the Pokot pastoralists in Kenya who had similar gender division of labor as our study group (i.e., men herded and women tended to household chores), men had longer total MVPA duration than women (Sayre, Pike, and Raichlen 2019). Gender differences in % total MVPA were detected among all participants but not when restricting to only non‐herders. In other words, men non‐herders and women engaged in similar daily percentages of total MVPA, but herders engaged in less percentage of total MVPA than women, after controlling for age and season. In this community, women were responsible for taking care of children, cooking multiple meals through the day, getting water, and doing the cleaning and washing—household activities that may have contributed to a higher percentage of total MVPA compared to men. Rural inhabitants tend to engage in more occupational and domestic PA than urban counterparts (Patterson et al. 2014), and this is especially true for women (Jayamani et al. 2013).

Gender differences in sleep were detected. We found that women had objectively longer and higher‐quality sleep (more efficient, less fragmented, lower percentage of nighttime wake bouts) than men, same as what was reported in Mong and Cusmano's (2016) review and several other field studies in subsistence populations (e.g., Kilius et al. 2021; McKinnon et al. 2022; Prall et al. 2018; Yetish, Kaplan, and Gurven 2018). Gender differences in sleep cannot be fully attributed to variation in daytime PA because low activity was the most consistent predictor for higher‐quality sleep, yet men and women did not significantly differ in their low activity durations. From conversations with participants, we know that in households with animals, men (including those who did not herd during the day) were responsible for getting up throughout the night to check on animals (e.g., cows, goats, sheep, horses). We also observed that men engaged in social activities later into the evening, such as visiting neighbors and taverns, attending gatherings, and preparing for funerals. We suspect that gender differences in nighttime responsibilities and social activities were key contributors to shorter and poorer quality sleep among men in this community; future work should examine these nighttime differences in more detail.

As predicted, the physical demands of constantly moving around with livestock were reflected in herders spending more percentage of their day in total low‐ and moderate‐to‐vigorous‐intensity activities and less in sedentary activity compared to non‐herders. Cattle post herders had higher percentage total MVPA than village herders and non‐herders. We observed that cattle post herders traveled greater distances throughout the day compared to village herders and non‐herders. Cattle post herders had to navigate the challenging topography of the vast mountainous landscape, walked farther to drive livestock to suitable pasture, and engaged in longer and more intensive activities (e.g., traveling up and down steep inclines) while patrolling the area to prevent animals from getting lost in forest patches and to protect them from wildlife. Future work should implement location tracking devices to quantify daily distance traveled by different occupation groups.

Interestingly, in our gender‐disaggregated models (Table S1), we did not find evidence of herders having worse sleep than non‐herders. While we found cattle post herders to have shorter sleep than non‐herders, village herders actually had higher sleep quality (higher SE, lower FI, lower % WASO) than non‐herders—muddling the idea that livestock presence was a key contributor to sleep disturbance. Residing in the village afforded more social opportunities such as frequenting taverns and visiting friends, as well as nighttime disturbances such as fear of crime (Hill et al. 2016) and noise from neighbors (i.e., population density is much higher in villages compared to cattle posts). As such, non‐herders may face similar levels of nighttime disturbances as village herders, with additional nighttime socializing and less burden for staying home to care for livestock—all contributing to non‐herders having poorer sleep than village herders.

### 4.3 Effects of Daytime Activity on Nighttime Sleep

Our results showed that total LPA and MVPA correlated with indicators of higher‐quality sleep (higher SE, lower FI, lower % WASO) among this agropastoral community who engaged in PA as part of their subsistence strategy. Our study contributes to the literature that PA can lead to better sleep (Kline et al. 2021; Kredlow et al. 2015; Wang and Boros 2021)—but *better* does not necessarily mean *longer*. Total LPA, total MVPA, and bouts of MVPA were associated with reduced TST. The opposing effects that LPA and MVPA had on sleep duration versus quality highlight the importance of examining these two measures as separate, but interconnected, features of sleep (Bin 2016). As sleep research progresses, the distinct effects of sleep duration and quality on health and well‐being are becoming increasingly evident and there is growing support for sleep quality, more so than duration, to promote health and well‐being. For example, studies have shown that sleep disturbance increased the risk for psychiatric disorders (Ford and Kamerow 1989), and that the quality of sleep, rather than quantity, predicted physiological risk factors for cardiovascular disease (Clark et al. 2016). Moreover, sleep disturbance was linked to an increased risk of developing type 2 diabetes (Anothaisintawee et al. 2016; Cappuccio et al. 2010). From a public health perspective, our findings offer strong support for prescribing PA as a way to maintain and improve health. From an evolutionary perspective, our findings corroborate results from phylogenetic analyses showing our species' proclivity to reduce overall TST in favor of much higher‐quality sleep compared to other primates (Nunn and Samson 2018).

We did not find evidence that longer durations of total MVPA resulted in longer sleep. We did not look at vigorous‐intensity activity separately due to methodological limitations that might have yielded inaccurate total and bouts of VPA (see Limitations). We urge future studies to disaggregate the effects of vigorous‐ versus moderate‐intensity activities on sleep as potential damage to the musculoskeletal system during sustained vigorous activity may require longer periods of rest, tapping into the restorative functions of sleep (Brinkman, Reddy, and Sharma 2023; Dijk 2009). Growth hormones, which are needed to stimulate protein synthesis for muscle growth, are secreted during sleep (Takahashi, Kipnis, and Daughaday 1968); thus, longer bouts of vigorous‐intensity activity may necessitate longer sleep duration (Dattilo et al. 2011; Kanaley et al. 1997; Lastella et al. 2018; Uchida et al. 2012).

### 4.4 Previous Night's Sleep Had No Effect on Daytime Activity

None of the sleep variables from the previous night (TST, FI, % WASO) had any significant association with daytime PA intensities. We suspect that in this study group, PA was the result of obligatory daily tasks (e.g., following the herd, cooking for family, planting, and harvesting crops) and must be tended to regardless of how well the individual slept the night before. PA stemming from these tasks are less compromisable than PA from leisure exercise, which is the main source of PA for those with more sedentary lifestyles in industrialized settings (Chennaoui et al. 2015; Wang and Boros 2021). The literature on the bidirectional relationship between daytime PA and nighttime sleep is scant, and we encourage researchers working with other groups (i.e., those with more flexibility in their daily PA) to explore the effects that previous night's sleep duration and quality may have on an individual's daytime PA intensities (Chennaoui et al. 2015).

Here, we showed that among habitually active individuals, sleep quality improved with PA at low intensity and less so at moderate‐to‐vigorous‐intensity activities. Our findings do not fully concur with recent reviews, which concluded that regular moderate‐intensity exercise was associated with longer and better sleep (Kline et al. 2021; Kredlow et al. 2015). We want to point out that in all the studies reviewed, PA was measured from purpose exercise. It could be that in this group where baseline activity was constant and already quite high, the total low‐intensity PA provided the necessary physiological benefits for promoting better sleep, such as decreasing cortisol and increasing HRV (Uchida et al. 2012). Pontzer's Constrained Total Energy Expenditure model illustrated the metabolic adaptions of habitually active individuals, where energy expenditure increased with PA at low‐intensity but plateaued at high‐intensity activity levels (Pontzer et al. 2015, 2016)—perhaps, a similar type of adaptation is at play in human sleep ecology. The physiological mechanisms underlying the differences between low‐, moderate‐, and vigorous‐intensity PA effects on sleep should be the focus of future studies.

## 5 Limitations

There were several study limitations that warrant discussion. First, we did not account for the type of activity or the bout of each activity; we only looked at the total daily duration and percentages of different PA intensities. An activity journal could be helpful for participants to record which activity they were partaking in (e.g., household chores, walking, traveling on horseback, herding, farming) and for how long. The type of sedentary behavior matters. For example, sitting for too long, more so than standing, was associated with an increased risk for cardiometabolic diseases (Hamilton et al. 2008), while certain sedentary postures (e.g., squatting) can conserve energy while maintaining lower limb muscle activation, thus offsetting the negative effects of sitting (Raichlen et al. 2020). A second limitation was that we had set all MotionWatch wristwatches to “Mode 1,” which means the device detected and recorded bodily acceleration along one axis. We chose “Mode 1” instead of “Mode 3” (tri‐axial detection) as it was the setting validated for sleep measures; we decided to forego the accuracy of PA measure for sleep measures. The PA levels reported in our study may not fully capture, and perhaps even undercount, participants' actual daytime activity levels. A third limitation was that we used MotionWare's default cut‐off points for delineating activity intensities. These cut‐off points were established from a cohort that did not lead as active of a lifestyle as the agropastoralists in this study; thus, the thresholds used for moderate‐ and vigorous‐intensity activities may have been too low. Future studies should have participants undergo an individual‐calibration test to determine more accurate cut‐off points. However, having lower thresholds for activity intensities may have ended up offsetting the undercounting of activity via uniaxial recording mode. Both the use of uniaxial mode and MotionWare's default cut‐off points for activity intensities were systematic error that affected all participants' PA data, and our analyses were restricted to within‐group comparisons; thus, we are still confident in the results reported in this paper. Furthermore, the total MVPA durations reported in this study were comparable to accelerometer‐derived PA in other subsistence groups (Appendix A, Table A2). Finally, we need to acknowledge that sleep is not a uniform state and is separated into rapid eye movement, or REM, and different stages of non‐REM, or NREM (Carskadon and Dement 2017). Sleep stages differ in their functions (Rechtschaffen 1998; Siegel 2005) and have been shown to respond differently to various PA intensities and durations (Kubitz et al. 1996; Youngstedt, O'Connor, and Dishman 1997). NREM sleep is most important for resting and restoration (Dijk 2009) while REM state, characterized by heightened brain activity, likely serves more neurocognitive functions (Siegel 2011). In this non‐invasive field study, we did not measure sleep architecture; thus, we can only speculate on the association between daytime PA and the total nighttime sleep duration and quality of the overall sleep bout.

## 6 Conclusion

Engaging in consistent low‐intensity activities was more beneficial for sleep than higher‐intensity activity among members of a rural agropastoral community living in the high‐altitude, mountainous region in Eastern Cape. All participants engaged in considerable amounts of daily non‐sedentary activities due to the physical demands of a remote rural living and the responsibilities of herding and farming subsistence lifestyles. We found that older participants engaged in less moderate‐to‐vigorous‐intensity activities and more in low‐intensity activities. Importantly, low‐intensity PA was the most consistent predictor for higher sleep quality. While herders, particularly cattle post herders, engaged in more non‐sedentary activities, only village herders were found to have better sleep quality than non‐herders. The relative effect of PA on sleep, while consistent and evident, is far from being the main driver for nighttime sleep variation in this community. Extrinsic factors, such as perceived safety (Hill et al. 2016; McKinnon, Shattuck, and Samson 2023) and local weather conditions (Samson et al. 2017; Yetish et al. 2015), can affect both sleep duration and quality. As global comparative data continue to reveal how much of our understanding of sleep is shaped by studies in the Global North, it becomes increasingly clear that we must consider the complex interactions among PA, environment, and cultural context to truly grasp sleep and activity variation across diverse human populations.

## Author Contributions

**Ming Fei Li:** conceptualization (lead); data curation (lead); formal analysis (lead); funding acquisition (supporting); investigation (lead); methodology (lead); project administration (supporting); visualization (lead); writing – original draft (lead); writing – review and editing (equal). **Puseletso Lecheko:** conceptualization (supporting); data curation (supporting); investigation (supporting); methodology (supporting); project administration (supporting); writing – review and editing (equal). **Tumelo Phuthing:** conceptualization (supporting); data curation (supporting); investigation (supporting); methodology (supporting); project administration (supporting); writing – review and editing (equal). **Tsepo Lesholu:** conceptualization (supporting); investigation (supporting); methodology (supporting); project administration (lead); writing – review and editing (equal). **David R. Samson:** conceptualization (supporting); funding acquisition (lead); investigation (supporting); methodology (supporting); project administration (supporting); resources (lead); supervision (lead); writing – review and editing (equal).

## Conflicts of Interest

The authors declare no conflicts of interest.

## Supporting information


**Appendix S1.** Supporting Information.

**Table S1.** Summary of LMMs on the effects of gender and occupation on daytime physical activity and nighttime sleep measures while controlling for age and season. Model 1 omits occupation as a predictor, Model 2 only included non‐herders, and Model 3 only included men. Significant effects (*p* < 0.05) are shown in bold. Abbreviations: NH is non‐herder, CPH is cattle post herder, VH is village herder, MVPA is moderate‐to‐vigorous‐intensity physical activity, LPA is low‐intensity physical activity, TST is total sleep time, SE is sleep efficiency, FI is fragmentation index, and WASO is wake after sleep onset.

## Data Availability

The full dataset used to generate the results of this study are available on request from the corresponding author. The raw data are not publicly available due to privacy or ethical restrictions. The individual‐level aggregate dataset is publicly available on OSF (https://osf.io/wk4xh/).
