# Supplementary material for: Low‐to‐Moderate Daytime Physical Activities Predicted Higher‐Quality Sleep Among Habitually Active Agropastoralists
Source: Am J Hum Biol. 2025 Feb 6;37(2):e70008. doi: 10.1002/ajhb.70008 (PMC11800054; doi:10.1002/ajhb.70008)
Supplement: Supplementary file 1 — Appendix S1. Supporting Information. [file AJHB-37-e70008-s001.docx]

Appendix A

**Actigraphy data processing and scoring sleep periods**

(A) Actogram of raw actigraphy data

**
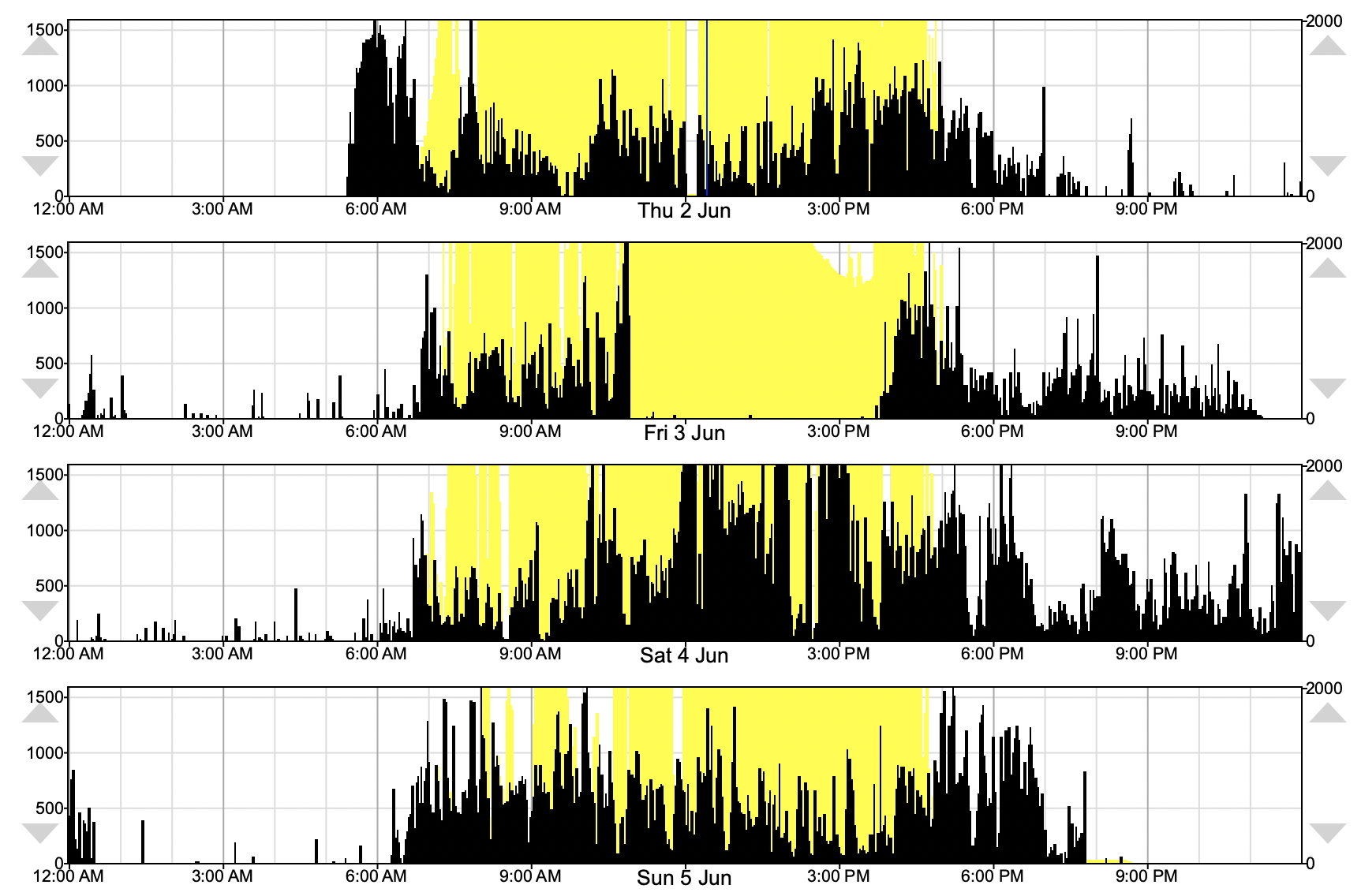
**

(B) Actogram of processed and scored actigraphy data

**
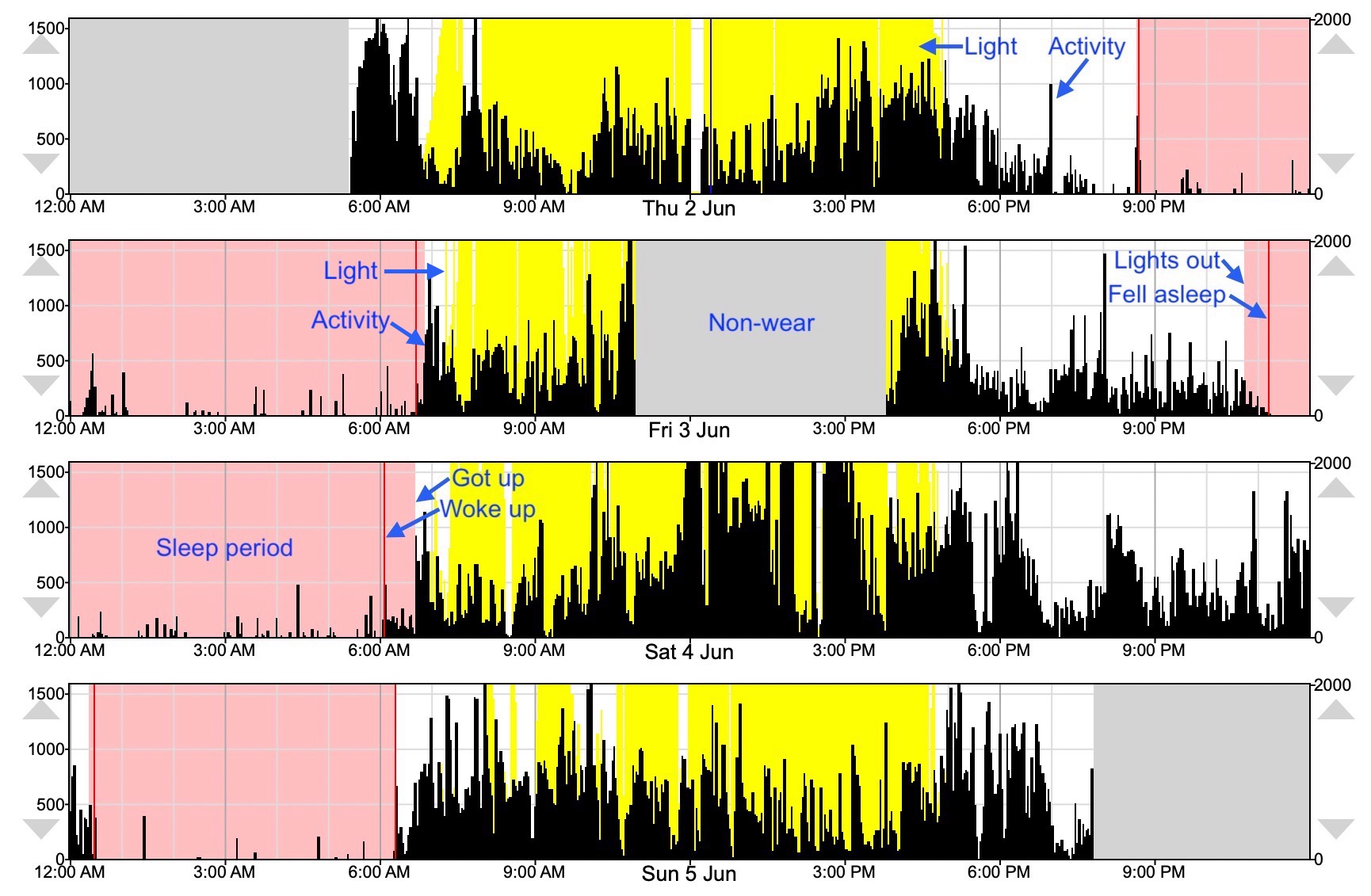
**

**Figure A1**. Actograms of (A) raw actigraphy data and (B) cleaned and processed data of a participant across two days. Each row spans 24 h, black bars are MotionWatch activity counts (left Y-axis), and yellow bars represent light (i.e., lux on right Y-axis) per one-minute epoch. In the processed file (B), the shaded red area represents the time in bed which is the duration between visually determined “light out” and “got up.” The red vertical lines within the shaded red area represents sleep onset and offset. Grey areas are excluded non-wear times.

**Table A1**. Definition of daytime physical activity and nighttime sleep measures (modified from the MotionWatch & MotionWatch User Guide: Issue 1.3.17a).

| Daytime Physical Activity Intensities | Definition |
| --- | --- |
| Total VPA: vigorous-intensity physical activity (h) | The total amount of time when the activity count exceeds the ‘Vigorous’ calibration threshold (1,000 counts per minute). |
| Total MPA: moderate-intensity physical activity (h) | The total amount of time when the activity count is greater than or equal to the ‘Moderate’ calibration threshold (500 counts per minute) and is less than ‘Vigorous’ threshold. |
| Total MVPA: moderate-to-vigorous-intensity physical activity (h) | The summation of Moderate and Vigorous Activity Times. |
| Total LPA: low-intensity physical activity (h) | The total amount of time when the activity count is greater than or equal to the ‘Sedentary calibration threshold (50 counts per minute) and is less than ‘Moderate threshold. |
| Sedentary activity (h) | The total amount of time when the activity count is greater than zero and is less than the ‘Sedentary’ threshold. |
| Bouts of VPA (h) | ‘Vigorous-intensity activity’ duration but only when the duration is 10 minutes or more. |
| Bouts of MPA (h) | ‘Moderate-intensity activity’ duration but only when the duration is 10 minutes or more. |
| Nighttime Sleep Measures | Definition |
| Time in bed (h) | The total elapsed time between the ‘Lights Out’ and ‘Got Up’ times. |
| Assumed sleep time (h) | The total elapsed time between the ‘Fell Asleep’ and ‘Woke Up’ times. |
| TST: Total sleep time (h) | The total time spent in sleep according to the epoch-by-epoch wake-sleep categorisation. |
| SE: Sleep efficiency (%) | Total sleep time expressed as a percentage of time in bed. |
| FI: Fragmentation index | The sum of the ‘Mobile time (%)’ and the ‘Immobile bouts <=1min (%)’ which are expressed as percentages of the assumed sleep time. This is an indication of the degree of fragmentation of the sleep period, and can be used as an indication of sleep quality (or the lack of it). |
| WASO: Wake after sleep onset (h) | The total time spent in wake according to the epoch-by-epoch wake-sleep categorisation. |
| % WASO (%) | WASO expressed as a percentage of the assumed sleep time. |

**Table A2.** Comparison of accelerometer-derived PA intensities in other small-scale subsistence groups

| **MVPA (min)** | **Group** | **Subsistence** | **Region** | **Reference** |
| --- | --- | --- | --- | --- |
| 201.75 | Basotho, Xhosa | Agropastoralist | South Africa | This study |
| 218.9 | Hadza | Hunter-gatherer | Tanzania | Sayre et al., 2020 |
| 200^a^ | Tsimane | Forager-horticulturalist | Bolivia | Gurven et al., 2013 |
| 188.52 | Pokot | Pastoralist | Kenya | Sayre et al., 2019 |
| 247.68^b^ | BaYaka | Forager | Congo Basin | Sarma et al., 2019 |

^a^ Estimate duration based on bar graph in Fig. 4

^b^ MVPA duration as calculated from 34.4% MVPA out of 12h wear time across one day
