# Supplementary material for: Low‐to‐Moderate Daytime Physical Activities Predicted Higher‐Quality Sleep Among Habitually Active Agropastoralists
Source: Am J Hum Biol. 2025 Feb 6;37(2):e70008. doi: 10.1002/ajhb.70008 (PMC11800054; doi:10.1002/ajhb.70008)
Supplement: Supplementary file 2 — Table S1. Summary of LMMs on the effects of gender and occupation on daytime physical activity and nighttime sleep measures while controlling for age and season. Model 1 omits occupation as a predictor, Model 2 only included non‐herders, and Model 3 only included men. Significant effects (p < 0.05) are shown in bold. Abbreviations: NH is non‐herder, CPH is cattle post herder, VH is village herder, MVPA is moderate‐to‐vigorous‐intensity physical activity, LPA is low‐intensity physical activity, TST is total sleep time, SE is sleep efficiency, FI is fragmentation index, and WASO is wake after sleep onset. [file AJHB-37-e70008-s002.docx]

Supporting Information for “Low to moderate daytime physical activities predicted higher quality sleep among habitually active agropastoralists”

**Table S1.** Summary of LMMs on the effects of gender and occupation on daytime physical activity and nighttime sleep measures, while controlling for age and season. Model 1 omits occupation as a predictor, Model 2 only included non-herders, Model 3 only included men. Signiﬁcant effects (P < 0.05) are shown in bold. Abbreviations: NH is non-herder, CPH is cattle post herder, VH is village herder, and MVPA is moderate-to-vigorous-intensity physical activity, LPA is low-intensity physical activity, TST is total sleep time, SE is sleep efficiency, FI is fragmentation index, WASO is wake after sleep onset

| Variable | β (SE) | P-value | β (SE) | P-value | β (SE) | P-value |
| --- | --- | --- | --- | --- | --- | --- |
| % Total MVPA | Model 1  N = 7,111, χ2 = 59.2, P < 0.001 | | Model 2  N = 3,133, χ2 = 34.8, P < 0.001 | | Model 3  N = 5,164, χ2 = 61.6, P < 0.001 | |
| Gender (ref: women) | - | - | - | - | - | - |
| Men | **-5.34 (1.37)** | **< 0.001** | -2.70 (2.04) | 0.191 | - | - |
| Occupation (ref: NH) | - | - | - | - | - | - |
| CPH | - | - | - | - | **3.19 (0.930)** | **< 0.001** |
| VH | - | - | - | - | **1.30 (0.650)** | **0.046** |
| Age | **-0.240 (0.040)** | **< 0.001** | **-0.379 (0.061)** | **< 0.001** | **-0.248 (0.043)** | **< 0.001** |
| Season (ref: summer) | - | - | - | - | - | - |
| Fall | **-1.75 (0.314)** | **< 0.001** | **-0.926 (0.445)** | **0.038** | **-1.67 (0.390)** | **< 0.001** |
| Winter | **-1.28 (0.270)** | **< 0.001** | -0.619 (0.389) | 0.111 | **-1.30 (0.331)** | **< 0.001** |
| % Total LPA | Model 1  N = 7,111, χ2 = 36.6, P < 0.001 | | Model 2  N = 3,133, χ2 = 30.6, P < 0.001 | | Model 3  N = 5,164, χ2 = 46, P < 0.001 | |
| Gender (ref: women) | - | - | - | - | - | - |
| Men | 1.91 (1.18) | 0.107 | -3.32 (1.97) | 0.097 | - | - |
| Occupation (ref: NH) | - | - | - | - | - | - |
| CPH | - | - | - | - | **1.98 (0.789)** | **0.012** |
| VH | - | - | - | - | 0.414 (0.564) | 0.463 |
| Age | **0.078 (0.034)** | **0.026** | **0.160 (0.059)** | **0.009** | **0.126 (0.033)** | **< 0.001** |
| Season (ref: summer) | - | - | - | - | - | - |
| Fall | -0.324 (0.280) | < 0.001 | -0.195 (0.421) | 0.644 | -0.169 (0.344) | 0.624 |
| Winter | **-1.25 (0.241)** | **< 0.001** | **-1.44 (0.368)** | **< 0.001** | **-1.29 (0.292)** | **< 0.001** |
| % Total Sedentary Activity | Model 1  N = 7,111, χ2 = 84.5, P < 0.001 | | Model 2  N = 3,133, χ2 = 30.9, P = 0.013 | | Model 3  N = 5,164, χ2 = 84.8, P < 0.001 | |
| Gender (ref: women) | - | - | - | - | - | - |
| Men | **3.20 (1.40)** | **0.024** | **6.01 (2.36)** | **0.013** | - | - |
| Occupation (ref: NH) | - | - | - | - | - | - |
| CPH | - | - | - | - | **-5.13 (1.03)** | **< 0.001** |
| VH | - | - | - | - | **-1.65 (0.723)** | **0.023** |
| Age | **0.171 (0.041)** | **< 0.001** | **0.216 (0.071)** | **0.003** | **0.125 (0.047)** | **0.010** |
| Season (ref: summer) | - | - | - | - | - | - |
| Fall | **2.09 (0.352)** | **< 0.001** | **1.12 (0.542)** | **0.038** | **1.81 (0.435)** | **< 0.001** |
| Winter | **2.55 (0.304)** | **< 0.001** | **2.05 (0.473)** | **< 0.001** | **2.56 (0.368)** | **< 0.001** |
| TST | Model 1  N = 7,111, χ2 = 259.9, P < 0.001 | | Model 2  N = 3,133, χ2 = 125, P < 0.001 | | Model 3  N = 5,164, χ2 = 163, P < 0.001 | |
| Gender (ref: women) | - | - | - | - | - | - |
| Men | **-0.450 (0.165)** | **0.007** | **-0.461 (0.210)** | **0.032** | - | - |
| Occupation (ref: NH) | - | - | - | - | - | - |
| CPH | - | - | - | - | **-0.327 (0.144)** | **0.023** |
| VH | - | - | - | - | 0.106 (0.104) | 0.308 |
| Age | **0.014 (0.005)** | **0.003** | 0.007 (0.006) | 0.253 | **0.013 (0.006)** | **0.024** |
| Season (ref: summer) | - | - | - | - | - | - |
| Fall | **0.418 (0.051)** | **< 0.001** | **0.366 (0.069)** | **< 0.001** | **0.421 (0.064)** | **< 0.001** |
| Winter | **0.681 (0.044)** | **< 0.001** | **0.666 (0.061)** | **< 0.001** | **0.651 (0.054)** | **< 0.001** |
| SE | Model 1  N = 7,111, χ2 = 27.7, P < 0.001 | | Model 2  N = 3,133, χ2 = 26.9, P < 0.001 | | Model 3  N = 5,164, χ2 = 35.5, P < 0.001 | |
| Gender (ref: women) | - | - | - | - | - | - |
| Men | -2.02 (1.188) | 0.090 | **-4.35 (1.486)** | **0.005** | - | - |
| Occupation (ref: NH) | - | - | - | - | - | - |
| CPH | - | - | - | - | 0.075 (0.892) | 0.933 |
| VH | - | - | - | - | **2.32 (0.617)** | **< 0.001** |
| Age | 0.050 (0.035) | 0.154 | 0.059 (0.045) | 0.189 | 0.071 (0.044) | 0.104 |
| Season (ref: summer) | - | - | - | - | - | - |
| Fall | **0.730 (0.286)** | **0.011** | **1.20 (0.387)** | **0.002** | **0.946 (0.368)** | **0.010** |
| Winter | **1.10 (0.247)** | **< 0.001** | **1.22 (0.338)** | **< 0.001** | **1.20 (0.311)** | **< 0.001** |
| FI | Model 1  N = 7,111, χ2 = 15.2, P = 0.004 | | Model 2  N = 3,133, χ2 = 16.1, P = 0.003 | | Model 3  N = 5,164 , χ2 = 10.2, P < 0.069 | |
| Gender (ref: women) | - | - | - | - | - | - |
| Men | **5.63 (1.65)** | **< 0.001** | **7.13 (2.28)** | **0.003** | - | - |
| Occupation (ref: NH) | - | - | - | - | - | - |
| CPH | - | - | - | - | -0.995 (1.39) | 0.474 |
| VH | - | - | - | - | **-2.13 (0.997)** | **0.033** |
| Age | **0.100 (0.048)** | **0.038** | 0.115 (0.069) | 0.097 | 0.095 (0.057) | 0.098 |
| Season (ref: summer) | - | - | - | - | - | - |
| Fall | 0.174 (0.485) | 0.719 | -0.638 (0.677) | 0.346 | -0.071 (0.612) | 0.907 |
| Winter | 0.745 (0.418) | 0.075 | 0.772 (0.590) | 0.191 | 0.570 (0.518) | 0.271 |
| % WASO | Model 1  N = 7,111, χ2 = 29.6, P = 0.004 | | Model 2  N = 3,133, χ2 = 28.9, P < 0.001 | | Model 3  N = 5,164, χ2 = 21.4, P < 0.001 | |
| Gender (ref: women) | - | - | - | - | - | - |
| Men | **2.58 (1.08)** | **0.017** | **4.29 (1.38)** | **0.003** | - | - |
| Occupation (ref: NH) | - | - | - | - | - | - |
| CPH | - | - | - | - | -0.604 (0.820) | 0.461 |
| VH | - | - | - | - | **-1.58 (0.569)** | **0.006** |
| Age | -0.032 (0.031) | 0.314 | -0.019 (0.042) | 0.655 |  |  |
| Season (ref: summer) | - | - | - | - | - | - |
| Fall | **-0.803 (0.264)** | **0.002** | **-1.30 (0.356)** | **< 0.001** | **-0.900 (0.340)** | **0.008** |
| Winter | **-0.982 (0.228)** | **< 0.001** | **-1.18 (0.310)** | **< 0.001** | **-1.01 (0.288)** | **< 0.001** |
